# The role of maternal DNA methylation in pregnancies complicated by gestational diabetes

**DOI:** 10.3389/fcdhc.2022.982665

**Published:** 2022-09-21

**Authors:** Stephanie Dias, Tarryn Willmer, Sumaiya Adam, Carmen Pheiffer

**Affiliations:** ^1^ Biomedical Research and Innovation Platform, South African Medical Research Council, Cape Town, South Africa; ^2^ Centre for Cardio-Metabolic Research in Africa, Division of Medical Physiology, Faculty of Medicine and Health Sciences, Stellenbosch University, Cape Town, South Africa; ^3^ Department of Obstetrics and Gynecology, Faculty of Health Sciences, University of Pretoria, Pretoria, South Africa; ^4^ Diabetes Research Center, Faculty of Health Sciences, University of Pretoria, Pretoria, South Africa

**Keywords:** DNA methylation, diabetes, pregnancy, gestational diabetes mellitus, type 1 diabetes mellitus, type 2 diabetes mellitus

## Abstract

Diabetes in pregnancy is associated with adverse pregnancy outcomes and poses a serious threat to the health of mother and child. Although the pathophysiological mechanisms that underlie the association between maternal diabetes and pregnancy complications have not yet been elucidated, it has been suggested that the frequency and severity of pregnancy complications are linked to the degree of hyperglycemia. Epigenetic mechanisms reflect gene-environment interactions and have emerged as key players in metabolic adaptation to pregnancy and the development of complications. DNA methylation, the best characterized epigenetic mechanism, has been reported to be dysregulated during various pregnancy complications, including pre-eclampsia, hypertension, diabetes, early pregnancy loss and preterm birth. The identification of altered DNA methylation patterns may serve to elucidate the pathophysiological mechanisms that underlie the different types of maternal diabetes during pregnancy. This review aims to provide a summary of existing knowledge on DNA methylation patterns in pregnancies complicated by pregestational type 1 (T1DM) and type 2 diabetes mellitus (T2DM), and gestational diabetes mellitus (GDM). Four databases, CINAHL, Scopus, PubMed and Google Scholar, were searched for studies on DNA methylation profiling in pregnancies complicated with diabetes. A total of 1985 articles were identified, of which 32 met the inclusion criteria and are included in this review. All studies profiled DNA methylation during GDM or impaired glucose tolerance (IGT), while no studies investigated T1DM or T2DM. We highlight the increased methylation of two genes, *Hypoxia‐inducible Factor‐3α (HIF3α)* and *Peroxisome Proliferator-activated Receptor Gamma-coactivator-Alpha (PGC1-α)*, and the decreased methylation of one gene, *Peroxisome Proliferator Activated Receptor Alpha (PPARα)*, in women with GDM compared to pregnant women with normoglycemia that were consistently methylated across diverse populations with varying pregnancy durations, and using different diagnostic criteria, methodologies and biological sources. These findings support the candidacy of these three differentially methylated genes as biomarkers for GDM. Furthermore, these genes may provide insight into the pathways that are epigenetically influenced during maternal diabetes and which should be prioritized and replicated in longitudinal studies and in larger populations to ensure their clinical applicability. Finally, we discuss the challenges and limitations of DNA methylation analysis, and the need for DNA methylation profiling to be conducted in different types of maternal diabetes in pregnancy.

## Introduction

Diabetes in pregnancy is associated with an increased risk of short- and long-term adverse outcomes, thus posing a serious health threat to both mother and offspring (1–3). The prevalence of diabetes in pregnancy is rapidly increasing and has been attributed to increasing maternal age and the rising rates of diabetes and obesity (4, 5). According to recent estimates, 21.1 million live births are affected by diabetes, of which a large portion, 80.3%, are due to gestational diabetes mellitus (GDM), a mild form of glucose intolerance that develops during pregnancy, 9.1% are due to type 1 (T1DM) or type 2 (T2DM) diabetes first detected in pregnancy and 10.6% are due to pregestational T1DM and T2DM (6). Diabetes during pregnancy has been associated with maternal (pre-eclampsia, cesarean deliveries, birth injury) and fetal (hyperbilirubinemia and polycythemia, macrosomia, large for gestational age, respiratory distress syndrome, congenital abnormalities, jaundice and perinatal mortality) adverse outcomes (7–9), while in the long-term both mothers and their babies have an increased risk of developing metabolic disease (10–12). Studies have reported that pregestational T1DM and T2DM are associated with more frequent and severe pregnancy complications compared to GDM. The more severe effects of pregestational diabetes on pregnancy are attributed to prolonged exposure to a hyperglycemic environment in the peri-conceptual period, exposure to an *in utero* hyperglycemic environment early during pregnancy and changes in placental structure and function, and the different pathophysiological mechanisms that underlie the different types of diabetes (13). A better understanding of the mechanisms that link the different types of diabetes in pregnancy with pregnancy complications may facilitate strategies to improve adverse pregnancy outcomes.

Epigenetics is defined as heritable alterations in gene expression that are not caused by changes in the DNA sequence (14). These processes include DNA methylation, histone and chromatin modifications, and non-coding RNAs that act as regulator molecules (15). DNA methylation is the most widely studied and best characterized epigenetic mechanism (16). It involves the covalent attachment of a methyl group to the fifth carbon position of a cytosine nucleotide to form 5-methylcytosine (5-mC). This process is catalyzed by the enzyme DNA methyltransferase (DNMT), with S-adenosyl-methionine serving as the methyl donor (16). DNA methylation mostly occurs on a cytosine base that precedes a guanine nucleotide (CpG site), which tend to cluster together to form CpG islands, and are primarily found within gene promoters, or in repeated elements such as long (LINE) and short (SINE) interspersed elements (17, 18). However, in recent years, studies have provided evidence of the importance of non-CpG and non-promoter methylation in the development of disease (19, 20). DNA methylation modifications regulate the transcriptional potential of the genome by inhibiting transcription factor binding, and is known to affect gene expression pathways associated with a range of pathophysiological processes, such as glucose and lipid homeostasis, insulin signaling and beta-cell function and, when dysregulated, contributes to metabolic disease (21–23).

DNA methylation has been shown to play a key role in regulating genes involved in metabolic adaptation during pregnancy, and aberrant DNA methylation has been demonstrated during pregnancy complications such as pre-eclampsia, hypertension, GDM, early pregnancy loss and preterm birth (24–27). Moreover, altered DNA methylation patterns have been observed in the placenta and cord blood of women with GDM, and have been identified as potential factors that mediate *in utero* fetal programming (26, 28–34). Thus, altered maternal DNA methylation patterns offer the potential to predict short- and long-term health complications in mothers and offspring exposed to an adverse intrauterine environment, such as hyperglycemia. This review aims to provide a summary of existing studies on DNA methylation in pregestational T1DM and T2DM, and GDM.

The inclusion criteria for this review included all studies reporting on DNA methylation profiling in women with T1DM, T2DM and GDM during pregnancy. Four databases, CINAHL, Scopus, PubMed and Google Scholar were searched to identify published studies that met the inclusion criteria No restrictions on dates were applied, and all articles until May 2022 were included. The following keywords, “pre-gestational diabetes”, OR “type 1 diabetes” OR “type 2 diabetes” OR “gestational diabetes mellitus” OR “maternal diabetes” OR hyperglycemia OR “hyperglycemia in pregnancy” OR “maternal glycemia” AND “DNA methylation” OR methylation OR epigenetics AND pregnancy OR antenatal OR prenatal OR maternal were used. Original articles profiling DNA methylation in women with diabetes in pregnancy and full-text articles published in English were included. The reference lists of included studies were searched to identify eligible articles that may have been missed in the search strategy.

## DNA methylation profiling in pregnancies complicated by diabetes

**Figure 1:**
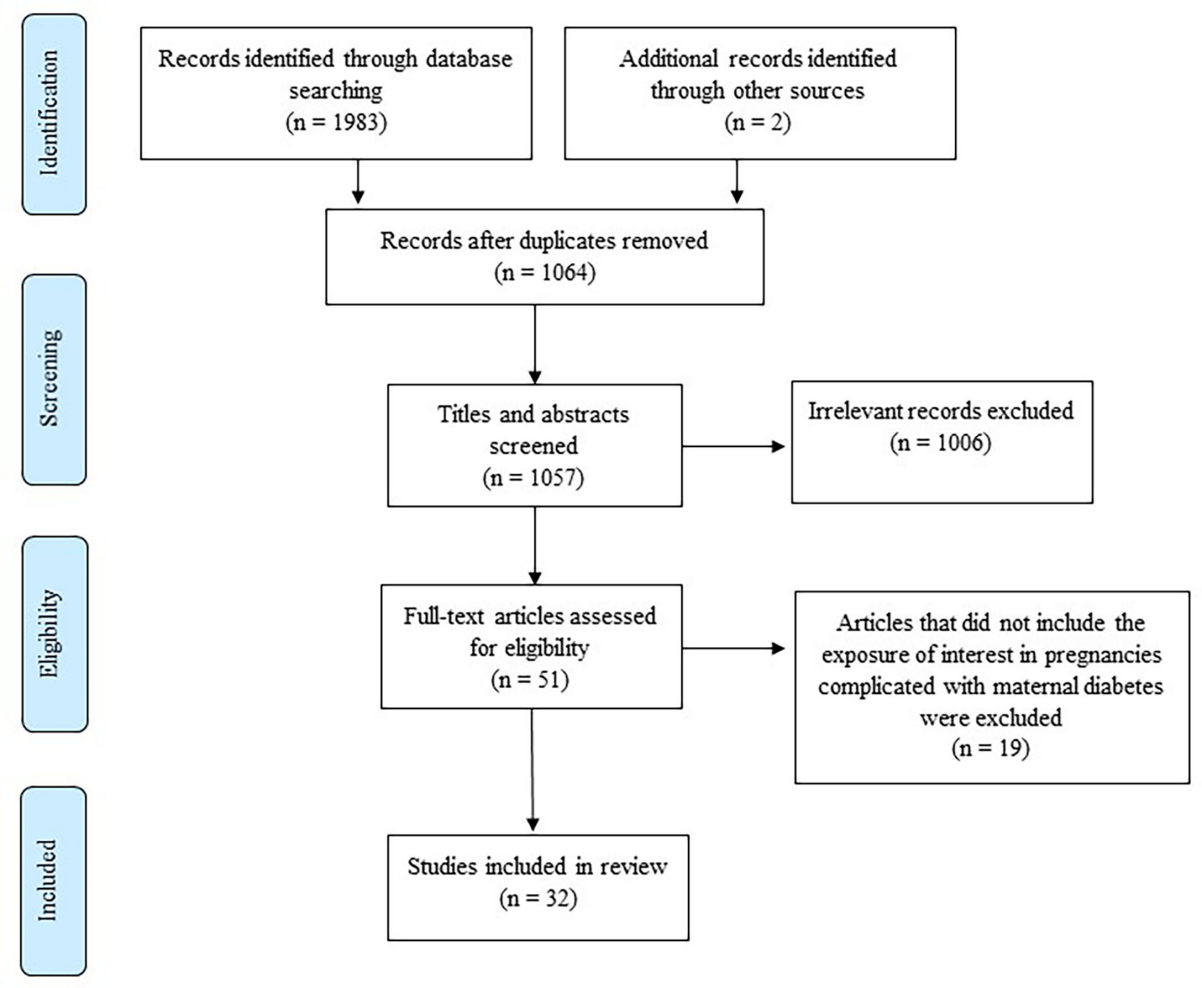
Flow diagram showing the selection of studies for inclusion in the review [adapted from (35)].

Our literature search identified a total of 1985 research articles, of which 32 met the inclusion criteria and are included in this review (**Figure 1**). The studies that investigated DNA methylation in pregnant women with diabetes are summarized in **Table 1**. Of the 32 studies, the majority investigated DNA methylation in women with GDM (n=28), two studies investigated DNA methylation in pregnant women with impaired glucose tolerance (IGT), and two studies investigated DNA methylation in pregnant women with both IGT and GDM groups. GDM is a widely recognized form of IGT that develops during pregnancy (59). Six studies diagnosed IGT or GDM using the World Health Organization (WHO), 1999 criteria (75g 2-hour oral glucose tolerance test (OGTT) ≥ 7.8 mmol/L), and although GDM and IGT may thus refer to the same condition, articles in this review are summarized according to the authors’ reporting, i.e., GDM or IGT. The studies included in this review used different diagnostic criteria, including the International Association of Diabetes in Pregnancy Study Group (IADPSG) (n=12), the German Society of Gynecology and Obstetrics guidelines (n=2), the American Diabetes Association (ADA) 2004 (n=1) and 2010 (n=1), Carpenter and Coustan (n=1), National Diabetes Data Group (n=1), WHO 1999 (n=4) and 2013 (n=1), both WHO 1999 and ADA 2009 (n=1) and WHO 1999 and IADPSG (n=1), a local criteria recommended by the Royal London Hospital, UK (n=1), and six studies did not report which diagnostic criteria were used (n=6). Of these, only four studies provided fasting plasma glucose (FPG), 1-hour and 2-hour OGTT values. Studies were conducted in various countries, and included Chinese (n=8), Canadian (n=8), German (n=3), South African (n=3), American (n=3), Taiwanese (n=2), European (n=2), South Asian (n=1), Japanese (n=1) and a mixed ethnic population (n=1). The sample size varied considerably between studies, ranging from six to 1030 women. DNA methylation was profiled in various biological materials, including maternal peripheral blood, omental visceral (VAT) and subcutaneous (SAT) adipose tissue, placenta (maternal and fetal side) and cord blood. The studies included in this review quantified DNA methylation using various approaches, including global DNA methylation (n=4), genome-wide methylation (n=12) and gene-specific methylation (n=22), which will be discussed in further detail. The included studies were case-control, cross-sectional or longitudinal studies.

**Table 1:** DNA methylation profiling in pregnancies complicated by diabetes.

#	Author (year)	Population	Sample size	GA	Diagnostic criteria	Method	Biological source	Type of diabetes	Treatment	Genes/region investigated	Study findings
** **	** **	** **	** **	** **	** **	** **	** **	** **	** **		** **
**1**	Awamleh et al. 2021 (28)	Canadian	Cord blood:	Delivery	National Diabetes Data group	Illumina HumanMethylation450	Cord blood and placenta	GDM	Cord blood:	Genome-wide methylation	Cord blood:
** **			N=42			BeadChip array			10 Diet,		99 differentially methylated CpG sites targeting 49 genes were identified
** **			16 GDM		100g 3hr OGTT				4 Insulin,		↓ 38.4%% CpG sites and
** **			26 Controls		FPG ≥ 5.8 mmol/L				2 unknown		↑ 61.6% CpG sits
** **					1 hr ≥ 10.6 mmol/L						
** **			Placenta:		2 hr ≥ 9.2 mmol/L				Placenta:		Placenta:
** **			N=27		3 hr ≥ 8.1 mmol/L				10 Diet,		662 differentially methylated CpG sites targeting 338 genes were identified
** **			11 GDM						6 Insulin,		↓ 75.2% CpG sites and
** **			16 Controls						4 unknown		↑ 24.8% CpG sits
** **											
** **											2 genes *AHRR* and *PTPRN2* overlapped between cord blood and placenta analyses
** **											
** **											The top biological processes were enriched for antigen processing and presentation via MHC class 1
** **											
**2**	Binder et al. 2015 (36)	American	N = 82	Delivery	Diagnostic criteria and values NR	*Discovery:*	Placenta	GDM	NR	Genome-wide methylation	***Placenta (maternal side):***
** **			41 GDM			Illumina HumanMethylation450 BeadChip array					
** **			41 controls							*CAPN1*	***Gene of interest (most significant) on array***
** **						*Verification and Validation:*					*↓* of *CAPN1*in the CpG locus within the intron
** **						Bisulfite pyrosequencing				4 locus selected close to candidate genes for verification and validation:	
** **										- *HLA-DOA, HLA-H/HLA-J, SNRPN/SNURF, CCDC181*	***4 locus selected:***
** **											↑ locus within an enhancer and 5′UTR of *CCDC181*
** **											↑ locus within the introns of *HLA-H*/*HLA-J*,
** **											↓ locus 285-bp upstream of the TSS of *HLA-DOA* and
** **											↓ locus within with the promoter of *SNRPN/SNURF* in women with GDM compared to pregnant women without GDM
** **											
** **											***Verification in same cohort:***
** **											↑ *CCDC181*
** **											↓ *HLA-DOA*
** **											↓ *SNRPN/SNURF*
** **											Trend towards significance for *HLA-HA*/*HLA-J*
** **											
** **											***Validation in independent cohort:***
** **											No significant difference observed
**3**	Bouchard et al. 2010 (37)	French-Canadian	N = 48	At delivery	WHO, 1999	Target sequencing combined with base specific cleavage	Placenta and cord blood	IGT	14 = Diet	Gene specific DNA methylation	***Placenta:***
** **			23 IGT						7 = Diet + Insulin		No significant difference observed between groups. Although *LEP* DNA methylation was correlated with 2hr glucose levels in IGT group
** **			25 Controls		75g 2hr OGTT				2 = no treatment	Leptin gene	
** **					2hr ≥7.8 mmol/l						***Cord blood:***
** **											↓ average CpG methylation of *LEP* in women with IGT compared to pregnant women with normoglycemia
**4**	Bouchard et al. 2012 (29)	French-Canadian	N = 100	At delivery	WHO, 1999 (IGT)	Bisulfite pyrosequencing	Placenta	IGT and GDM	17 = Diet	Gene specific DNA methylation	***Placenta (fetal side):***
** **			31 IGT						14 = Diet +Insulin		
** **			67 Controls		75 g 2 hr OGTT					*ADIPOQ* locus (3 CpG islands, 17 CpGs and mean)	Average ↓ of *ADIPOQ at* C1 (CpG1-4) and E2mean1 (CpG 3) in pregnant women with IGT compared to normoglycemia
** **			2 GDM		FPG < 7.0 mmol/L						
** **					2 hr ≥ 7.8 mmol/L						No significant difference observed for *ADIPOQ* E2mean2
** **											
** **					ADA, 2009 (GDM)						
** **											
** **					75g 2hr OGTT						
** **					FPG ≥ 5.3 mmol/L						
** **					1 hr ≥ 10 mmol/L						
** **					2 hr ≥ 8.6 mmol/L						
**5**	Chen et al. 2021 (38)	Chinese	N = 46	At delivery	IADPSG	Methylation specific PCR	Placenta	GDM	NR	Gene specific DNA methylation	***Placenta (maternal side):***
** **			23 GDM								*↑ of MEG3* at *7* CpGs and average overall methylation in women with GDM compared to pregnant women without GDM
** **			23 controls		75g 2 hr OGTT					*MEG3* locus (35 CpGs)	
** **					FPG ≥ 5.1 mmol/L						Increased MEG3 was correlated with maternal hyperglycemia, neonatal birthweight and was associated with decreased gene expression
** **					1 hr ≥ 10.0 mmol/L						
** **					2 hr ≥ 8.5 mmol/L						
** **											***Placenta (fetal side):***
** **											No significant difference for *MEG3* methylation and mRNA expression was observed
** **											
**6**	Cộtộ et al. 2016 (39)	Canadian	*0*	At delivery	IADPSG	Bisulfite pyrosequencing	Placenta	GDM	NR	Gene/locus specific methylation	↓ of average *BMP7 in women* with GDM compared to pregnant women without GDM
** **											
** **			N = 133		75g 2 hr OGTT					*PRDM16, BMP7, CTBP2*,and *PGC-1α* gene loci	Trend towards ↑ of 2 CpG sites within *PPARγC1α* which was correlated with glucose levels in the second trimester, and associated with cord blood leptin levels in offspring
** **			33 GDM		FPG ≥ 5.1 mmol/L						
** **			100 Controls		1 hr ≥ 10 mmol/L						No significance in *CTBP2* and *PRDM16*
** **					2 hr ≥ 8.5 mmol/L						
**7**	Deng et al. 2018 (40)	Chinese	N = 50	At delivery	WHO, 2013	*Discovery:*	Omental VAT	GDM	NR	Genome-wide methylation	5910 differentially methylated regions targeting 1298 genes which were ↓, whereas
** **			26 GDM			Illumina HumanMethylation450					6892 differentially methylated regions targeting 1568 genes were ↑
** **			24 controls		75g 2hr OGTT	BeadChip				7 candidate genes	
** **					FPG ≥ 5.1mmol/L					overlapping between DEGs and DMGs were selected:	Of the seven candidate genes overlapping between DEGs and DMGs only *MSLN* showed typical negative correlation between gene expression and methylation
** **					1hr ≥ 10.0 mmol/L	*Verification:*				*C10orf10, FSTL1, GSTT1, HLA-DPB1, HLA-DRB5, HSPA6* and *MSLN*	
** **					2hr ≥ 8.6mmol/L	Bisulfite pyrosequencing					
** **											Functional analysis of differentially methylated genes revealed pathways mostly enriched for graft-versus-host disease, type I diabetes mellitus, antigen processing and presentation and allograft rejection
**8**	Desgagnộ et al. 2014 (41)	Canadian	2 cohorts:	Delivery	WHO, 1999 (IGT)	Bisulfite pyrosequencing	Placenta	IGT	*ECO21*	Gene specific DNA methylation	↓ *IGF1R* and *IGFBP3* in women with IGT compared to pregnant women with normoglycemia in the ECO21 birth cohort
** **									19 = Diet		
** **			*0*		75 g 2 hr OGTT				15 = Diet + Insulin		↓ *IGF1R* observed in women with IGT and GDM compared to pregnant women with normoglycemia in the Gen-3G birth validation cohort, while
** **			N = 140		2 hr ≥ 7.8 mmol/L					*IGF1R* and *IGFBP3*	no significance for *IGFBP3* was observed
** **			34 IGT								
** **			106 Controls		IADPSG (GDM)						
** **											
** **			*- Gen-3G*		75g 2 hr OGTT						
** **			N = 30		FPG ≥ 5.1 mmol/L						
** **			11 IGT		1 hr ≥ 10 mmol/L						
** **			4 GDM		2 hr ≥ 8.5 mmol/L						
** **			15 Controls								
**9**	Dias et al. 2019 (44)	South African	N = 24	<26 weeks gestation	IADPSG	Illumina HumanMethylationEPIC BeadChip array	Peripheral blood	GDM	NR	Genome-wide methylation	1046 differentially methylated CpG sites corresponding to 939 genes were observed in women with GDM compared pregnant women without GDM.
** **			12 GDM								↑ of 148 CpG sites (14.2%) and ↓ of 898 CpG sites (85.8%) were observed
** **			12 Controls		75g 2 hr OGTT						
** **					FPG ≥ 5.1 mmol/L						Functional analysis revealed a significant association with pathways such as cancer, brain signaling, cell growth, proliferation, viability and inflammatory pathways.
** **					1 hr ≥ 10 mmol/L						
** **					2 hr ≥ 8.5 mmol/L						
**10**	Dias et al. 2019b (43)	South African	N = 201	<26 weeks gestation	IADPSG	Imprint global methylation DNA quantification (ELISA)	Peripheral blood	GDM	NR	Global DNA methylation	No difference in global DNA methylation in women with GDM compared to pregnant women without GDM.
** **			63 GDM								
** **			138 controls		75g 2 hr OGTT						↑ Global methylation in obese compared to non-obese women was observed.
** **					FPG ≥ 5.1 mmol/L						
** **					1 hr ≥ 10.0 mmol/L						Moreover, ↑ global methylation was associated with ↓ serum adiponectin levels
** **					2 hr ≥ 8.5 mmol/L						
**11**	Dias et al. 2021 (44)	South African	N = 286	<26 weeks gestation	IADPSG	Bisulfite pyrosequencing	Peripheral blood	GDM	NR	Gene specific DNA methylation	↓ at CpG -3400 in *ADIPOQ* in women with GDM compared to pregnant women without GDM
** **			95 GDM								
** **			191 Controls		75g 2 hr OGTT					*ADIPOQ*	DNA methylation at CpG -3400 was positively associated fasting glucose and negatively associated with serum adiponectin levels
** **					FPG ≥ 5.1 mmol/L						
** **					1 hr ≥ 10 mmol/L						
** **					2 hr ≥ 8.5 mmol/L						
**12**	El Hajj et al. 2013 (30)	German	N= 251	Delivery	Diagnostic Criteria NR	Bisulfite pyrosequencing	Placenta and cord blood	GDM	88 = Diet	Global DNA methylation	***Placenta:***
** **			88 D-GDM						98 = Insulin		↓ Global DNA methylation of Alu and ↑ LINE1 repetitive elements methylation in women with GDM compared to pregnant women without GDM
** **			98 I-GDM		75g 2 hr OGTT					*ALU and LINE1 repeats*	
** **			65 controls		FPG > 5.3mmol/L						↓*MEST, PPARα, NR3C1* and *NESPAS* in women with GDM compared to pregnant women without GDM
** **					1 hr > 10.0mmol/L					and	
** **					2 hr > 8.6mmol						No significance observed for other candidate genes
** **										Gene specific methylation	
** **											***Cord blood:***
** **										*Imprinted genes*	↓ Global methylation Alu and LINE1 repetitive elements methylation in women with GDM compared to pregnant women without GDM
** **										*#VALUE!*	
** **										*-LIT1, MEST, NESPAS, PEG3, SNRPN*	*↓ MEST, NR3C1, OCT4, NDUFB6* (D-GDM), methylation
** **										*Metabolic genes*	*↑ IL10, NDUFB6* (I-GDM), LINE1 methylation in women with GDM compared to pregnant women without GDM
** **										*-LEP, NDUFB6, NR3C, PPARα*	
** **										*Anti-inflammatory gene*	No significance observed for other candidate genes. Although *MEG3* methylation differed between male and female cord blood samples
** **										*0*	
** **										*Tumor suppressor gene*	
** **										*-APC*	
** **										*Pluripotency gene*	
** **										*0*	
**13**	Enquobahrie et al. 2015 (45)	American	N = 6	<20 weeks gestation	ADA, 2004	Illumina HumanMethylation27	Peripheral blood	GDM	NR	Genome-wide methylation	27 differentially methylated CpG sites identified between GDM and normal pregnancies within the same women. Of these, 17 CpG sites were hypomethylated and 10 CpG sites were hypermethylated
** **			3 GDM			BeadChip array					
** **			3 controls		100g 3hr OGTT					Candidate genes commonly methylated in participants	***Candidate genes identified:***
** **					1hr ≥ 10mmol/L						↓ *NDUFC1, HAPLN3*, *HHLA3*,
** **					2hr ≥ 8.6mmol/L					- *NDUFC1, HAPLN3, HHLA3, RHOG*	and *RHOG* and ↑ *SEP11, ZAR1*, and *DDR* between GDM and normal pregnancies. Candidate genes were associated with gene pathways such as cell cycle, cell morphology, cell assembly, cell organization, and cell compromise
** **					3hr ≥ 7.8mmol/L					*SEP11, ZAR1, and DDR*	
** **											
**14**	Finer et al. 2015 (31)	South Asian	Cord blood	Delivery	Local diagnostic criteria	Illumina HumanMethylation450	Cord blood and placenta	GDM	74% = Diet	Genome-wide methylation	***Cord blood:***
** **			N = 49			BeadChip array			19% = Insulin or metformin		1418 methylated variable positions (β-value difference >5%) were identified in women with GDM compared to pregnant women without GDM
** **			27 GDM		75g 2hr OGTT					No candidate genes identified	
** **			21 controls		FPG ≥ 5.8 mmol/L and/or						***Placenta:***
** **					2hr ≥7.8mmol/L						1373 methylated variable positions (β-value difference >5%) were identified in women with GDM compared to pregnant women without GDM
** **			Placenta:								
** **			N = 43								387 methylated variable positions were common in both cord blood and placenta
** **			25 GDM								
** **			18 controls								Functional analysis revealed gene pathways enriched endocytosis, focal adhesion, chemokine signaling and ligand receptor interactions
**15**	Haertle et al. 2017 (46)	Middle and Southeastern European	N = 313	At delivery	IADPSG	*Discovery:*	Cord blood	GDM	105 = Insulin	Genome-wide methylation	1564 differentially methylated CpG sites identified.
** **						Illumina HumanMethylation450 BeadChip array			88 = Diet		
** **			105 I-GDM *(10-17% had T1D or T2D before pregnancy)*		75g 2 hr OGTT					4 Candidate genes selected from the methylation array	Using a more stringent criteria, 65 differentially methylated CpG sites associated with 52 genes were identified in women with I-GDM compared to pregnant women without GDM.
** **			88 D-GDM		FPG ≥ 5.1 mmol/L	*Verification:*					
** **			120 Controls		1 hr ≥ 10 mmol/L	Bisulfite				-*ATP5A1, MFAP4, PRKCH, SLC17A4*, and *HIF3A* (selected due to its correlation with BMI)	No significance in women with D-GDM
** **					2 hr ≥ 8.5 mmol/L	pyrosequencing					
** **											***Candidate genes:***
** **											*↓ ATP5A1 at CpG2 and PRKCH at CpG1-3*
** **											*↑* of *HIF3A* promoter at *CpG5-6* and *CpG10-11, SLC17A4* CpG2 in women with I-GDM and D-GDM compared to pregnant women without GDM.
** **											
** **											no significant difference in *MFAP4*
**16**	Houde et al. 2013 (32)	Canadian	N = 100	Delivery	WHO, 1999 (IGT)	Bisulfite pyrosequencing	Placenta,	IGT	Women with IGT:	Gene specific DNA methylation	***Placenta (maternal and fetal side):***
** **			26 IGT				Peripheral blood and cord blood		13 = Diet		No significant *ABCA1* methylation differences were observed in women with IGT compared to pregnant women with normoglycemia
** **			74 Controls		75 g 2 hr OGTT				12 = Diet + Insulin	*ABCA1* gene locus (8 CpG sites and mean methylation)	
** **					2 hr ≥ 7.8 mmol/L				1 = unknown		***Peripheral blood:***
** **											Mean *↓* of *ABCA1* in women with IGT compared to pregnant women with normoglycemia
** **											
** **											***Cord blood:***
** **											A trend towards significance for mean *↓* of *ABCA1* in women with IGT compared to pregnant women with normoglycemia
** **											
**17**	Houde et al. 2014 (47)	Canadian	N = 126	Delivery	WHO, 1999	Bisulfite pyrosequencing	Placenta	GDM	16 = Diet	Gene specific DNA methylation	***Placenta (fetal side):***
** **			27 GDM						11 = Diet + Insulin		↓ of the LPL proximal promoter region at CpG1 and intron 1 CpG island (CpG sites 2 and 3) in women with GDM compared to pregnant women without GDM
** **			99 Controls		75 g 2 hr OGTT					*LPL* gene locus (3 CpG sites)	
** **					2 hr ≥ 7.8 mmol/L						
**18**	Kang et al. 2017 (48)	Taiwanese	N = 16	Delivery	IADPSG	Illumina HumanMethylationEPIC BeadChip array	Peripheral blood and cord blood	GDM	NR	Genome-wide methylation	***Peripheral blood:***
** **			8 GDM								The top 200 loci selected corresponded to 151 differentially methylated genes in women with GDM
** **			8 Controls		75g 2 hr OGTT					No candidate genes selected	
** **					FPG ≥ 5.1 mmol/L						***Cord blood:***
** **					1 hr ≥ 10 mmol/L						The top 200 loci corresponded to 167 differentially methylated genes in women with GDM
** **					2 hr ≥ 8.5 mmol/L						
** **											Functional analysis revealed an association with pathways enriched for endocrine disorders, metabolic diseases, carbohydrate metabolism, lipid metabolism, as well JAK2/STAT-3 and MAPK signaling
**19**	Kang et al. 2018 (49)	Taiwanese	N = 32	At delivery	IADPSG	Methylation specific PCR	Peripheral blood, cord blood and placenta	GDM	Diet	Gene specific DNA methylation	***Peripheral blood:***
** **			8 GDM								↓ of IL-10 in women with GDM compared to pregnant without GDM
** **			24 Controls		75g 2 hr OGTT					*IL-10*	
** **					FPG ≥ 5.1 mmol/L						↓ of IL-10 was associated with increased serum IL-10 concentrations.
** **					1 hr ≥ 10.0 mmol/L						
** **					2 hr ≥ 8.5 mmol/L						***Cord blood:***
** **											No significant difference
** **											
** **											***Placenta:***
** **											No significant difference
**20**	Kasuga e al. 2022 (50)	Japanese	N= 230	Delivery	2 step	Illumina HumanMethylationEPIC BeadChip array	Cord blood	GDM	Diet + Insulin	Genome-wide methylation	754 255 CpG sites investigated showed no methylation differences between women with GDM compared to pregnant women without GDM
** **			167 GDM		50g 1hr glucose				(number of participants not specified)		
** **			63 controls		1hr ≥ 7.8mmol/L						
** **											
** **					IADPSG						
** **											
** **					75g 2 hr OGTT						
** **					FPG ≥ 5.1 mmol/L						
** **					1 hr ≥ 10 mmol/L						
** **					2 hr ≥ 8.5 mmol/L						
**21**	Nomura et al. 2014 (26)	American	N = 50	Delivery	Carpenter and Coustan	Lumino-metric Methylation Assay	Placenta and cord blood	GDM	NR	Global DNA methylation	***Placenta:***
** **			8 GDM								↓ Global DNA methylation in women with GDM compared to pregnant women without GDM
** **			42 controls		100g 3 hr OGTT						
** **					FPG > 5.3 mmol/L						***Cord blood:***
** **					1hr ≥ 10.0 mmol/L						No differences in global DNA methylation in cord blood in women with GDM compared to pregnant women without GDM
** **					2hr ≥ 8.6 mmol/L						
** **					3hr ≥ 7.8 mmol/L						
**22**	Ott et al. 2018 (51)	German	N = 55	At delivery	German Society of Gynecology and Obstetrics guidelines	Bisulfite pyrosequencing	SAT, VAT, Peripheral blood and cord blood	GDM	13 = Diet and/or Insulin	Gene specific DNA methylation	***SAT:***
** **			25 GDM								↑ R2 CpG2 *ADIPOQ*
** **			30 Controls		75g 2hr OGTT					10 CpGs in the *ADIPOQ* gene locus	No significance in R1 and R3
** **					FPG ≥ 5.0 mmol/L						
** **					1 hr ≥ 10 mmol/L						***VAT:***
** **					2 hr ≥ 8.6 mmol/L						↑ R3 CpG1 *ADIPOQ*
** **											↓ R1 CpG4 *ADIPOQ*
** **											No significance in R2
** **											An inverse correlation between DNA methylation and mRNA expression at specific CpG sites in R2 and R3 across both SAT and VAT.
** **											
** **											***Peripheral blood:***
** **											↑ R1 CpG1 *ADIPOQ*
** **											↑ R2 mean *ADIPOQ*
** **											↑ R2 CpG4 *ADIPOQ*
** **											
** **											***Cord blood:***
** **											↓ R2 CpG1-4 *ADIPOQ*
** **											↑ R3 CpG1-4 *ADIPOQ*
** **											No significance observed in R1
** **											
**23**	Rancourt et al. 2021 (52)	German	N = 41	Delivery	German Society of Gynecology and Obstetrics guidelines	Bisulfite pyrosequencing	Omental VAT and Peripheral blood	GDM	NR	Gene specific DNA methylation	***VAT:***
** **			19 GDM								↓ of *SOCS3* at CpG5-6 within exon 2 in women with GDM compared to pregnant women without GDM
** **			22 Controls		75g 2hr OGTT					*SOCS3*	
** **					FPG ≥ 5.0 mmol/L						***Peripheral blood:***
** **					1 hr ≥ 10 mmol/L						No significant difference observed for all CpG sites
** **					2 hr ≥ 8.6 mmol/L						
**24**	Reichetzeder et al. 2016 (53)	Mixed ethnicity (Germany)	N = 1030	Delivery	IADPSG	LC-MS/MS	Placenta	GDM	NR	Global DNA methylation	↑ Global DNA methylation in placenta of women with GDM compared to pregnant women without GDM
** **			56 GDM								
** **			974 controls		75g 2 hr OGTT						
** **					FPG ≥ 5.1 mmol/L						
** **					1 hr ≥ 10.0 mmol/L						
** **					2 hr ≥ 8.5 mmol/L						
**25**	Rong et al. 2015 (54)	Chinese	N = 76	At delivery	ADA, 2010	*Discovery:*	Placenta	GDM	19 = Diet	Gene/locus specific methylation	6 641 DMRs identified targeting 3320 genes, of which 2 729 showed significant hypermethylation and 3 912 DMRs targeting 1970 genes showed significant hypomethylation in women with GDM compared to pregnant women without GDM
** **			36 GDM			MeDIP microarray			17 = Diet + Insulin		
** **			40 controls		75g 2hr OGTT					Specific CpGs on array	***Validated candidate genes:***
** **						*Verification:*					↓ of *GLUT3, Resistin, and PPARα* in women with GDM compared to women without GDM
** **					FPG ≥ 5.3 mmol/L	Bisulfite pyrosequencing				Candidate genes selected due its role in molecular mechanism underlying GDM:	
** **					1hr ≥ 10.0 mmol/L						*PPARα* was upregulated in GDM group, although, no significance was observed
** **					2hr ≥ 8.6 mmol/L					*GLUT3, Resistin, RBP4 and PPARα*	
** **											No significance for *RBP4*
**26**	Ruchat et al. 2013 (33)	Canadian	N = 44	At delivery	WHO, 1999	Illumina HumanMethylation450 BeadChip array	Cord blood and placenta	GDM	16=Diet	Genome-wide methylation	***Cord blood:***
** **			30 GDM						14=Diet + Insulin		CpG sites correlated to 3758 differentially methylated genes in women with GDM compared to pregnant women without GDM
** **			14 Controls		75 g 2 hr OGTT						
** **					2 hr ≥ 7.8 mmol/L						***Placenta:***
** **											CpG sites correlated to 3271 differentially methylated genes in women with GDM compared to pregnant women without GDM
** **											
** **											25% (1029) of differentially methylated genes were common in both tissues, and were associated with glucose-metabolism related pathways
** **											
**27**	Wang et el. 2018 (55)	Chinese	N = 40	At delivery	Diagnostic criteria NR	Direct methylation sequencing	Placenta	GDM	NR	Gene/locus specific methylation	***Placenta (fetal side):***
** **			20 GDM								
** **			20 controls		2 step						↑ methylation of *PPARγC1α* which correlated with decreased gene expression levels in women with GDM compared to pregnant women without GDM
** **					50g 1hr glucose					*PPARγC1α (PGC-1α) and PDX1* (7 CpGs)	
** **					1hr ≥ 7.8mmol/L						*↑* methylation of *PDX1*, although not significance
** **											
** **					100g glucose						
** **					FPG > 5.6mmol//l, 1hr > 10.3mmol/L, 2hr > 8.6mmol/L, 3hr > 6.7mmol/L						
**28**	Wu et al. 2018 (56)	European	N = 22	12-16 weeks of gestation	Diagnostic criteria and values NR	*Discovery:*	Peripheral blood	GDM	NR	Genome-wide methylation	100 differentially methylated CpGs correlated to 66 genes
** **			11 GDM			Illumina HumanMethylation450 BeadChip array					
** **			11 controls							Top 5 candidate genes with the highest significance	***Verification (in 8 of 11 women):***
** **						*Verification:*					*COPS8, PIK3R5, HAAO, CCDC124,and C5orf34* genes (no mention of hypermethylation or hypomethylation)
** **						Bisulfite				- *COPS8, PIK3R5, HAAO, CCDC124,and C5orf34*	
** **						pyrosequencing					
**29**	Xie et al. 2015 (34)	Chinese	N = 58	At delivery	IADPSG	Bisulfite	Placenta and cord blood	GDM	NR	Gene/locus specific methylation	***Placenta (fetal side):***
** **			24 GDM			pyrosequencing					↑ *of PGC-1α* promoter region in women with GDM compared to pregnant women without GDM
** **			34 Controls		75g 2 hr OGTT					*PGC-1α* promoter	
** **					FPG ≥ 5.1 mmol/L 1 hr ≥ 10 mmol/L						***Cord blood:***
** **					2 hr ≥ 8.5 mmol/L						↓ of *PGC-1α* promoter region, which was correlated with higher maternal glucose levels in women with GDM compared to pregnant women without GDM
** **											
**30**	Yan et al. 2021 (57)	Chinese	N = 239	At delivery	IADPSG	*Discovery:*	Cord blood	GDM	NR	Genome-wide methylation	1251 genes differentially methylated in women with GDM compared to pregnant women without GDM
** **			107 GDM			Illumina HumanMethylation450					
** **			132 Controls		75g 2 hr OGTT	BeadChip array				Candidate gene selected based on number of significant CpGs:	***Validation:***
** **					FPG ≥ 5.1 mmol/L						↓CpG sites (cg12604331, cg08480098) in the gene body of *ARHGEF11*, which was negatively correlated with neonatal outcomes and birthweight
** **					1 hr ≥ 10 mmol/L	*Validation:*				*ARHGEF11*	
** **					2 hr ≥ 8.5 mmol/L	Mass spectrometry combined with base specific cleavage					
**31**	Zhang et al. 2019 (23)	Chinese	N = 40	During surgical intervention (GA NR)	Diagnostic criteria NR	Bisulfite pyrosequencing	Omental tissue	GDM	NR	Gene specific DNA methylation	↑ of the 2 CpG Island within *HIF3A* promoter in women with GDM compared to pregnant women without GDM. DNA methylation was negatively correlated with gene expression levels
** **			20 GDM								
** **			20 Controls		FPG ≥ 5.5mmol/L					*HIF3A*	
** **					1h ≥ 10mmol/L						
** **					2h ≥ 8.6mmol/L						
**32**	Zhao et al. 2019 (58)	Chinese	N = 30	Delivery	Diagnostic criteria NR	Methylation specific PCR	Placenta	GDM	NR	Gene specific DNA methylation	***Placenta (maternal side):***
** **			15 GDM								↑ of *DLK1* at 9 CpG sites and mean methylation in women with GDM compared to pregnant women without GDM
** **			15 Controls							*DLK1* locus (38 CpG sites located in proximal promoter)	
** **					75g 2hr OGTT						***Placenta (fetal side):***
** **					FPG ≥ 5.0mmol/L						↑ of *DLK1* at 3 CpG sites, while no mean *DLK1* methylation differences were observed in women with GDM compared to pregnant women without GDM
** **					1hr ≥ 10mmol/L						
** **					2hr ≥ 6.2mmol/L						

Several genes such as, Adiponectin (ADIPOQ), Suppressor of Cytokine Signaling 3 (SOCS3), Hypoxia Inducible Factor 3 Subunit Alpha (HIF3A), Peroxisome Proliferator-activated Receptor Gamma Coactivator 1-alpha (PGC-1α), PR Domain Containing 16 (PRDM16), Bone Morphogenetic Protein 7 (BMP7), C-Terminal Binding Protein 2 (CTBP2), H19 Imprinted Maternally Expressed Transcript (H19), Maternally Expressed 3 (MEG3), Long QT Intronic Transcript 1 (LIT1), Mesoderm Specific Transcript (MEST), Paternally Expressed 3 (PEG3), Small Nuclear Ribonucleoprotein Polypeptide N (SNRPN), SNRPN Upstream Open Reading Frame (SNURF), Leptin (LEP), NADH:Ubiquinone Oxidoreductase Subunit B6 (NDUFB6) and C1 (NDUFC1), Nodal Homolog 3-C (NR3C), Peroxisome Proliferator Activated Receptor Alpha (PPARα), Interleukin-10 (IL-10), adenomatous polyposis coli (APC), Organic Cation/Carnitine Transporter4 (OCT4), ATP Binding Cassette Subfamily A Member 1 (ABCA1), Lipoprotein Lipase (LPL), Solute Carrier Family 9 Member A3 (SLC9A3), Male-Enhanced Antigen 1;Kelch Domain-Containing Protein 3 (MEA1;KLHDC3), Calmodulin Binding Transcription Activator 1 (CAMTA1), RAS P21 Protein Activator 3 (RASA3), Collectin Subfamily member 10 (COLECT10), Rho Guanine Nucleotide Exchange Factor 11 (ARHGEF11), decidual protein induced by progesterone 1 (C10orf10/DEPP1), Follistatin-like 1 (FSTL1), Glutathione S-transferase theta 1 (GSTT1), HLA Class II Histocompatibility Antigen, DRB5 Beta Chain (HLA-DRB5), Heat Shock Protein Family A (Hsp70) Member 6 (HSPA6), Mesothelin (MSLN), Constitutive Photomorphogenic Homolog Subunit 8 (COPS8), Phosphoinositide-3-Kinase Regulatory Subunit 5 (PIK3R5), 3-Hydroxyanthranilate 3,4-Dioxygenase (HAAO), Coiled-Coil Domain Containing Protein 124 (CCDC124), Chromosome 5 Open Reading Frame 34 (C5orf34), ATP Synthase F1 Subunit Alpha (ATP5A1), Microfibril-Associated Glycoprotein 4 (MFAP4), Protein Kinase C Eta Type (PRKCH), Solute Carrier Family 17 Member 4 (SLC17A4), Hypoxia Inducible Factor 3 Subunit Alpha (HIF3A), Hyaluronan And Proteoglycan Link Protein 3 (HAPLN3), HERV-H LTR-Associating 3 (HHLA3), Ras Homology Growth-Related (RHOG), Septin 11 (SEP11), Zygote Arrest 1 (ZAR1), Discoidin Domain Receptor (DDR), Calpain 1 (CAPN1), Major Histocompatibility Complex, Class II, DO Alpha (HLA-DOA), Major Histocompatibility Complex, Class I, H/J (HLA-H/HLA-J), Coiled-Coil Domain Containing 181 (CCDC181), Glucose Transporter 3 (GLUT3), Resistin (RETN), Retinol Binding Protein 4 (RBP4), Delta Like Non-Canonical Notch Ligand 1 (DLK1), and Pancreatic and Duodenal Homeobox 1 (PDX1), Aryl Hydrocarbon Receptor Repressor (AHRR) and Protein Tyrosine Phosphatase Receptor Type N2 (PTPRN2), Subcutaneous Adipose Tissue (SAT), Visceral Adipose Tissue (VAT), Gestational Diabetes Mellitus (GDM), Gestational Age (GA), Not Reported (NR), World Health Organization (WHO), American Diabetes Association (ADA), International Association of Diabetes in Pregnancy (IADPSG), Oral Glucose Tolerance Test (OGTT), Fasting Plasma Glucose (FPG), Polymerase Chain Reaction (PCR), Liquid Chromatography Mass Spectrometry (LC-MS/MS).

### Global DNA methylation studies

Global DNA methylation is a measure of the overall genomic methylation and is one of the earliest changes associated with the development of disease (60). Current methods to quantify global DNA methylation include direct methods such as enzyme-linked immunosorbent assays (ELISAs), liquid chromatography coupled with mass spectrometry (LC-MS/MS), high-performance capillary electrophoresis and methylation-sensitive restriction enzymes, and surrogate methods that quantify DNA methylation within repetitive elements as a marker of global DNA methylation (61). The repetitive elements LINE-1 and SINE-1 (mainly Alu) are highly represented throughout the genome and methylation of these elements have been used as a surrogate marker of global genomic DNA methylation. These repetitive elements are quantified using bisulfite pyrosequencing (62).

Four studies quantified global DNA methylation in women with GDM (**Table 1**). Dias et al. quantified global DNA methylation in the peripheral blood of 201 South African women with or without GDM using the Imprint Global DNA methylation ELISA (43). These authors reported no difference in global DNA methylation in the peripheral blood of women with GDM compared to pregnant women without GDM. Interestingly, this study showed higher levels of global DNA methylation in pregnant women who were obese compared to pregnant women who were not obese. Furthermore, these authors demonstrated that higher levels of global DNA methylation were associated with lower serum adiponectin levels (43), an important adipokine previously shown to be inversely associated with insulin resistance during pregnancy (63, 64). Nomura et al. measured global DNA methylation in the cord blood and placenta of 50 pregnant women with or without GDM from the USA using the Lumino-Metric methylation assay (26). The authors reported no difference in global DNA methylation levels in the cord blood of women with GDM compared to pregnant women without GDM, while lower levels of methylation were observed in the placenta of women with GDM compared to women without GDM (26). Reichetzeder et al. measured global DNA methylation in the placenta of 1030 pregnant women with mixed ethnic ancestry from Germany using LC-MS/MS. These authors reported higher levels of global DNA methylation in the placenta of women with GDM compared to pregnant women without GDM (53). El Hajj et al. assessed methylation of repetitive elements in the placenta and cord blood of 251 pregnant German women using bisulfite pyrosequencing. The authors demonstrated lower LINE-1 and Alu methylation in the cord blood of women with GDM compared to pregnant women without GDM, while an increase in LINE-1 methylation and decrease in Alu methylation was observed in the placenta of women with GDM compared to pregnant women without GDM (30). Taken together, these findings highlight the variability in assessing global DNA methylation levels in different biological sources such peripheral blood, cord blood and placenta, and using different methods of quantification.

### Gene-specific methylation studies

Measurement of global DNA methylation is inexpensive and robust, yet does not have the resolution to detect DNA methylation differences within specific genes (65). The quantification of gene-specific methylation at individual CpG sites may elucidate the role of DNA methylation in regulating the expression of genes that orchestrate the development of disease. As such, gene-specific DNA methylation is increasingly being used to identify genes associated with diabetes in pregnancy. Methods to quantify gene-specific DNA methylation include bisulfite pyrosequencing, methylation-specific PCR, methylated DNA immunoprecipitation (MeDIP), direct methylation sequencing and target sequencing combined with base-specific cleavage (61).

Twenty two studies investigated gene-specific DNA methylation in pregnant women with GDM or IGT (**Table 1**), of which, five studies were gene-specific validation studies for genome-wide DNA methylation quantification, using BeadChip Arrays (36, 40, 46, 56, 57). Of the 22 studies, a total of 62 genes were investigated across the studies. Of these, eight of the genes investigated overlapped in two or more populations and are discussed below. These genes included adiponectin (*ADIPOQ)*, Hypoxia Inducible Factor 3 Subunit Alpha *(HIF3α)*, Interleukin-10 *(IL-10)*, Leptin *(LEP)*, Maternally Expressed 3 *(MEG3)*, Peroxisome Proliferator-activated Receptor Gamma Coactivator 1-alpha *(PGC-1α)*, Peroxisome Proliferator Activated Receptor Alpha *(PPARα*) and Small Nuclear Ribonucleoprotein Polypeptide N *(SNRPN)*.

The three studies that quantified DNA methylation of *ADIPOQ* in women with GDM or IGT reported conflicting results (29, 44, 51). Dias et al. quantified DNA methylation at eight CpG sites located upstream of the *ADIPOQ* transcription start site (TSS) in the peripheral blood of 286 South African women with or without GDM using bisulfite pyrosequencing (44). These authors reported decreased methylation at one CpG site in the distal promoter region located -3400bp upstream of the TSS in women with GDM compared to women without GDM. Interestingly, the authors showed that DNA methylation levels were positively correlated with fasting glucose concentrations and negatively correlated with serum adiponectin concentrations (44). In line with these findings, Bouchard et al. reported lower placental DNA methylation at two CpG islands, located in the proximal promoter and between the first and second exon of *ADIPOQ* in 100 French-Canadian women with IGT compared to pregnant women with normoglycemia using bisulfite pyrosequencing (29). In contrast to these studies, Ott et al. demonstrated increased methylation levels at majority of the investigated CpG sites in VAT, SAT, maternal blood and cord blood of German women with GDM using bisulfite pyrosequencing (51). In SAT, DNA methylation at one CpG site located upstream of the TSS within the proximal promoter of *ADIPOQ* was higher in women with GDM compared to women without GDM, while DNA methylation in VAT revealed both higher and lower methylation at one CpG site located between exon 1 and 2 and one in the proximal promoter region of *ADIPOQ*, respectively. Moreover, in SAT and VAT, gene expression of *ADIPOQ* was significantly lower in women with GDM compared to women without GDM and was positively correlated with maternal circulating adiponectin levels. CpG sites investigated in SAT and VAT, were similarly hypermethylated in the maternal blood of women with GDM compared to women without GDM, while CpG sites located in the proximal promoter region or between exon 1 and 2 within *ADIPOQ* were either hypo- and hypermethylation in cord blood (51). *ADIPOQ* encodes an adipose tissue-derived hormone that plays a key role in whole-body energy homeostasis and is involved in regulating glucose and lipid metabolism and insulin sensitivity (66). Diabetes in pregnancy is characterized by insulin resistance, which increases as pregnancy progresses. Accordingly, lower adiponectin concentrations are observed with pregnancy duration (63, 64) and *ADIPOQ* levels are decreased in women with GDM compared to pregnant women without GDM (67). Taken together, findings from these studies showed that *ADIPOQ* methylation levels at specific CpG sites in SAT and VAT are correlated with lower *ADIPOQ* gene expression and circulating adiponectin levels, supporting the functional relevance in GDM.

Both studies profiling DNA methylation of *HIF3α* reported increased methylation in the omental tissue and cord blood of women with GDM compared to women without GDM (23, 46). Zhang et al. evaluated the methylation status of *HIF3α* in the omental tissue of Chinese women using bisulfite pyrosequencing and identified increased methylation at two CpG islands within the *HIF3α* promoter in women with GDM compared to women without GDM (23). In addition, the authors demonstrated that *HIF3α* promoter methylation was negatively correlated with *HIF3α* gene expression. Similarly, using bisulfite pyrosequencing, Haertle et al. reported that the average methylation levels of 11 CpG sites in the *HIF3α* promoter region were significantly higher in cord blood of European women with insulin and diet treated GDM compared to women without GDM (46). *HIF3α* is a member of the transcription factor family of hypoxia‐inducible factors, which are known to regulate a wide range of target genes related to glucose and amino acid metabolism, adipocyte differentiation, inflammation and cancer (68, 69). Recent studies have shown that *HIF3α* methylation is associated with adipose tissue dysfunction and insulin sensitivity, which are key factors contributing to GDM pathogenesis (70, 71). Considering the increased *HIF3α* promoter methylation in omental tissue and cord blood and its correlation with *HIF3α* gene regulation, these findings highlight the potential of *HIF3α* methylation as a candidate biomarker for novel therapeutic targets for GDM treatment.

The two studies quantifying DNA methylation of *IL-10* in the cord blood, placenta and peripheral blood of women with GDM reported conflicting results (30, 49). Using bisulfite pyrosequencing, El Hajj et al. quantified DNA methylation of *IL-10* in 251 German women with or without GDM. The authors reported an increase in *IL-10* promoter methylation in the cord blood of women with GDM compared to women without GDM, while no significant difference were observed in placenta (30). Conversely, using methylation specific PCR, Kang et al. reported decreased *IL-10* methylation in the peripheral blood of 32 Taiwanese women who developed GDM compared to women without GDM, while no significant differences were observed in the cord blood and placenta (49). Furthermore, the authors showed that decreased methylation of *IL-10* was associated with increased serum *IL-10* concentrations at the end of pregnancy. *IL-10* is an anti-inflammatory cytokine, which plays an important role in regulating the innate immune system (72), and has been associated with inflammatory-associated diseases such as obesity and diabetes (73). Recently, both increased and decreased *IL-10* serum levels have been reported to be associated with diabetes and GDM (74–76). Accordingly, findings from these studies demonstrate tissue and cell type specific *IL-10* methylation differences and suggest that lower methylation of *IL-10* at specific CpGs may play an important role in the development of GDM but needs to be explored in larger prospective studies to confirm its association.

Two studies investigating DNA methylation of *LEP* in the cord blood and placenta of French-Canadian and German women with GDM or IGT reported conflicting results (30, 37). Bouchard et al. assessed DNA methylation at 31 CpG sites within the *LEP* proximal promoter, using targeted sequencing combined with base specific cleavage. The authors reported a decrease in average CpG methylation in the cord blood of French-Canadian women with IGT compared to pregnant women with normoglycemia, while no significant differences were observed in the placenta (37). Interestingly, the authors demonstrated that average placental methylation at the *LEP* promoter CpG sites was significantly correlated with 2-hour OGTT glucose concentrations in women with IGT compared to pregnant women with normoglycemia. Another study using bisulfite pyrosequencing showed no significant difference in both the placenta and cord blood of German women with GDM (30). However, this study only assessed six of the 31 CpG sites investigated by Bouchard. *LEP* encodes an adipocytokine that is involved in energy metabolism and insulin sensitivity control, and is expressed and secreted by the placenta during pregnancy (77). Accordingly, several studies have shown that *LEP* expression and plasma levels are increased in obesity, diabetes and GDM, as well as during pregnancy (78–80). Taken together, these findings provide evidence that *LEP* DNA methylation may be influenced by glucose dysregulation during pregnancy, although further longitudinal studies are required to confirm whether *LEP* DNA methylation is a cause or consequence of GDM.

The two studies investigating DNA methylation of *MEG3* in women with GDM showed conflicting results, with one study reporting increased methylation in the placenta of Chinese women, and the other reporting no significant change in the placenta and cord blood of German women with GDM (30, 38). Chen et al. assessed 35 CpG sites located within *MEG3* in 46 Chinese women with or without GDM, using methylation specific PCR (38). The authors demonstrated increased maternal placental methylation at seven CpG sites within *MEG3* in women with GDM compared to women without GDM, which was correlated with maternal hyperglycemia and neonatal birthweight. Furthermore, the authors demonstrated that increased methylation on the maternal side of the placenta was associated with decreased *MEG3* gene expression, whereas no *MEG3* methylation and gene expression changes were observed on the fetal side of the placenta (38). Using bisulfite pyrosequencing, Hajj et al. showed no difference in placenta and cord blood methylation at three CpG sites located in the *MEG3* promoter and five CpG sites located in the *MEG3* intergenic region of 251 German women with GDM compared to women without GDM. However, *MEG3* methylation differed significantly between male and female cord blood samples regardless of GDM. *MEG3* is a maternally expressed imprinting gene, produced by long non-coding RNA transcripts that is regulated by two differentially methylated regions (DMRs) (81, 82). Thus, results from these studies suggest that DNA methylation modifications of the imprinting gene *MEG3* could potentially regulate *MEG3* gene expression during GDM and may play an essential role in fetal development in response to an intrauterine hyperglycemic environment.

Three studies investigating DNA methylation of *PGC-1α* reported increased placental methylation in Chinese and French-Canadian women with GDM (34, 39, 55), while one of these studies also reported decreased *PGC-1α* methylation in cord blood of Chinese in Chinese women with GDM (34). Xie et al. quantified DNA methylation in the *PGC-1α* promoter region in Chinese women with GDM, using bisulfite pyrosequencing (34). The authors reported increased placental methylation and decreased cord blood methylation in women with GDM compared to women without GDM. Similarly, Côté et al. showed a trend towards increased placental methylation of *PGC-1α* which was correlated with higher second trimester glucose levels in French-Canadian women with GDM compared to women without GDM, using bisulfite pyrosequencing (39). In addition, these authors showed that increased methylation levels of one CpG site within *PGC-1α* significantly correlated with cord blood leptin levels in the offspring. Using direct methylation sequencing, Wang et al. reported an increase in DNA methylation of *PGC-1α* on the fetal side of the placenta in Chinese women with GDM compared women without GDM, which was significantly correlated with decreased *PGC-1α* gene expression levels (55). *PGC-1α* is a transcriptional co-activator, which plays an important regulatory role in mitochondrial biogenesis and function, oxidative stress and insulin resistance, implicating *PGC-1α* in the development of glucose intolerance (83, 84). Accordingly, *PGC-1α* methylation and mRNA expression has been shown to be altered in diabetic compared to non-diabetic patients (85). Altogether, these results indicate that increased *PGC-1α* methylation is associated with glucose intolerance during pregnancy and could potentially influence gene regulatory pathways involved in developmental programming and offspring health outcomes.

Two studies investigating DNA methylation of *PPARα* reported decreased placental methylation in women with GDM (30, 54), while no significant difference was observed in cord blood (30). Rong et al. evaluated the methylation status of *PPARα* in the placenta of Chinese women and reported decreased methylation levels at 2 CpG sites within the promoter region in women with GDM compared women without GDM, using bisulfite pyrosequencing (54). Moreover, the authors reported that the expression of *PPARα* was upregulated in the GDM group compared to controls, although, these results were not significant. In a German population, El Hajj et al. reported decreased average placental methylation across 8 CpG sites within the *PPARα* promoter (30), while no significant difference was observed in the cord blood of women with GDM compared to women without GDM, using bisulfite pyrosequencing. *PPARα* is a ligand-activated transcriptional factor, known to regulate the expression of genes involved in fatty acid oxidation, and has been shown to be associated with energy metabolism in pregnancy (86), and when dysregulated, may be involved in the molecular mechanisms postulated to underlie GDM. Thus, these findings demonstrate aberrant DNA methylation patterns of *PPARα* methylation in GDM which may be involved in the pathophysiology of GDM and reflect fetal development. However, future studies are required to assess the therapeutic potential of these findings.

Two studies quantified DNA methylation of *SNRPN* in women with GDM, and reported conflicting results (30, 36). Binder et al. reported decreased placental methylation of *SNRPN* in American women with GDM compared to women without GDM, which was validated in a larger sample size, using a BeadChip array and bisulfite pyrosequencing, respectively (36). However, no significant methylation differences were observed when validating these results in an independent cohort, using bisulfite pyrosequencing. Similarly, using bisulfite pyrosequencing, El Hajj et al. reported no significant *SNRPN* methylation differences in the cord blood and placenta of German women with GDM compared to women without GDM (30). *SNRPN* is a maternally imprinted gene, which is related to various neurodevelopmental disorders (87), and the lack of *SNRPN* expression has been linked to hyperphagia, loss of satiety, and obesity (88, 89). Furthermore, imprinted genes such as *SNRPN* are essential for the regulation of human fetal and placental growth (90), and abnormal methylation of *SNRPN* has been reported in several imprinting syndromes (91, 92). While studies linking *SNRPN* to GDM are still lacking, these findings demonstrate a potential role of altered placental methylation of *SNRPN* during GDM that needs to be explored in future studies.

Other articles in this review reported differential methylation of genes, yet these genes were identified in single studies only (32, 41, 52, 58, 93).

### Genome-wide methylation studies

Due to rapid technological advances, genome-wide DNA methylation profiling has emerged as most popular platform for DNA methylation analysis. Genome-wide methylation strategies allow for a comprehensive, high-throughput quantitative approach to assess the methylation status of CpG sites for the entire genome (94). The platform provides an unbiased approach to identify both known and novel methylation sites. The techniques used to assess genome-wide methylation include various Illumina BeadChip Arrays such as the HumanMethylation27, HumanMethylation450 and the HumanMethylationEPIC array, as well as various methylation sequencing platforms such as Sanger or capillary sequencing, next-generation sequencing, whole genome bisulfite sequencing, methylated DNA immunoprecipitation, methylation sensitive restriction enzyme and Methyl-CpG-binding domain protein capture sequencing (95).

In this review, 12 studies quantified genome-wide DNA methylation using different Infinium methylation BeadChip arrays. Of the 12 studies, three studies used the HumanMethylationEPIC BeadChip, while eight studies used the older HumanMethylation450 BeadChip and one study used the HumanMethylation27 BeadChip array. Although these arrays use the same technology, they differ in the range of genomic coverage (27,000 to 850,000 CpG sites across the genome) and may lead to the identification of distinct methylation profiles (94, 96). In one of the earliest genome-wide studies, using the Illumina HumanMethylation27 BeadChip array, which interrogates approximately 27,000 CpG sites across the genome at a single-nucleotide resolution, Enquobahrie et al. reported DNA methylation changes in the peripheral blood of six American women who had two consecutive pregnancies, one of which was complicated by GDM during early pregnancy (45). The authors reported differential methylation at 27 CpG sites, of which 17 CpG sites were hypomethylated, and 10 CpG sites were hypermethylated between GDM and normal pregnancies within the same women. Novel genes related to these CpG sites were found to be associated with the cell cycle, cell morphology, cell assembly, cell organization, and cell compromise (45). These findings suggest that DNA methylation differences in peripheral blood reflect changes in GDM status in women with repeat pregnancies.

Eight of the 12 studies investigated in this review used the Illumina HumanMethylation450 BeadChip Array, which interrogates more than 480,000 methylation sites and covers 96% of CpG islands, as well as additional island shores (94). Ruchat et al. showed that CpG sites corresponding to 3271 genes in the placenta and 3758 genes in the cord blood were differentially methylated in Canadian women with GDM compared to women without GDM. Of these, 1029 differentially methylated genes were common to both tissues (33). *In silico* analysis revealed that these differentially methylated genes are predominantly involved in metabolic disease pathways, including glucose metabolism-related disorders which were amongst the top ranked pathways in both placenta and cord blood. Finer et al. identified 1418 methylated variable positions in cord blood and 1373 methylated variable positions in the placenta of South Asian women with GDM compared to women without GDM (31). Of these, 378 methylated variable positions were common to cord blood and placenta. Functional analysis of these methylated variable positions in placenta, cord blood and those overlapping both tissue, revealed gene pathways enriched in endocytosis, focal adhesion, chemokine signaling and ligand receptor interactions. Furthermore, these pathways display methylation differences in genes involved in key extracellular triggers to a myriad of intracellular signaling pathways involved in growth and metabolism (31). Haertle et al. reported differential methylation at 1564 CpG sites in the cord blood of middle and Southeastern European women with GDM compared to women without GDM (46). Using more robust statistical analysis, the authors reported the differential methylation of 65 CpG sites that were associated with 52 genes in women with insulin dependent GDM (I-GDM) compared to women without GDM, while no difference was observed in women with dietetically treated GDM (D-GDM) compared to women without GDM. In another study, Deng et al. quantified genome-wide DNA methylation in omental VAT of Chinese women with GDM. The authors reported differential methylation of 5910 regions targeting 1298 genes which were hypomethylated, whereas 6892 differentially methylated regions targeting 1568 genes were hypermethylated in women with GDM compared to women without GDM (40). Functional analysis showed that the differentially methylated genes were association with graft-versus-host disease, T1DM, antigen processing and presentation and allograft rejection biological pathways. Wu et al. identified 100 differentially methylated CpG sites corresponding to 66 genes in the peripheral blood of European women with GDM compared to women without GDM (56). Using a more stringent criteria to prioritize methylation sites, five CpG sites within the *constitutive photomorphogenic homolog subunit 8* (*COPS8*), *phosphoinositide 3-kinase regulatory subunit 5* (*PIK3R5*), *3-hydroxyanthranilate 3,4-dioxygenase* (*HAAO*), *coiled-coil domain containing 124* (*CCDC124*), and *chromosome 5 open reading frame 34* (*C5orf34*), were identified and validated using bisulfite pyrosequencing. Methylation differences of these CpG sites were detected early during pregnancy and may prove useful as predictive biomarkers of GDM. However, this study included 11 women with and without GDM only, and thus, requires further validation in a larger sample size (56). Yan et al. identified 1251 differentially methylated genes in the cord blood of Chinese women with GDM compared to women without GDM (57). The authors demonstrated decreased methylation at two CpG sites in the gene body of *Rho Guanine Nucleotide Exchange Factor 11* (*ARHGEF11)*, a key gene associated with metabolic pathways, which was negatively correlated with glucose levels and neonatal birth weight (57). Using a stringent criteria, Binder et al. reported the differential methylation of 648 CpG loci in the placenta of women with GDM compared to women without GDM in an American population. Functional analysis of genes in close proximity to the differentially methylated CpG loci revealed that the most significantly enriched biological processes where associated with cellular metabolism, response to external stimuli, and immune responses (36). More recently, Awamleh et al. showed that 99 CpG sites corresponding to 49 genes in cord blood, and 662 CpG sites corresponding to 338 genes in placenta were differentially methylated in Canadian women with GDM compared to women without GDM (28). Two of these genes, *aryl hydrocarbon receptor repressor (AHRR)* and *protein tyrosine phosphatase receptor type N2 (PTPRN2)*, were differentially methylated in both cord blood and placental samples. Functional analysis of the differentially methylated genes revealed that the top biological pathways were enriched for antigen processing and presentation *via* MHC class 1, which plays a vital role in regulating immune responses. Together, these findings indicate that differentially methylated genes, identified in the placenta, cord blood, adipose tissue, and peripheral blood of women with GDM are involved in several metabolic processes that may play an important role in the development of GDM. However, future studies should consider validation or verification of these differentially methylated genes in different populations to confirm their feasibility.

Three of the 12 studies included in this review quantified DNA methylation using the most recent Illumina HumanMethylationEPIC BeadChip array, which interrogates over 850 000 CpG sites across the genome at single-nucleotide resolution (94, 96). Dias et al. investigated DNA methylation in the peripheral blood of South African women with and without GDM, and reported differential methylation of 1046 CpG sites targeting 939 genes in women with GDM compared to women without GDM (42). Of these, 148 CpG sites (14.2%) were hypermethylated, while the 898 CpG sites (85.8%) were hypomethylated in women with GDM compared to women without GDM. Functional enrichment analysis of the differentially methylated genes revealed a significant association with pathways such as cancer, brain signaling, cell growth, proliferation, viability, and inflammatory pathways. Kang et al. showed that 200 CpG sites corresponding to 151 genes in peripheral blood and 167 genes in cord blood were differentially methylated in Taiwanese women with GDM compared to women without GDM (48). Functional analysis of differentially methylated genes demonstrated an association with pathways linked to endocrine disorders, metabolic diseases, carbohydrate metabolism, and lipid metabolism, as well as metabolically related signaling pathways such as Janus kinase 2/signal transduction and activator of transcription 3 *(JAK2/STAT-3)* and mitogen activated protein kinase *(MAPK)* in women with GDM compared to women without GDM. Conversely, in a more recent study, Kasuga et al. quantified DNA methylation at 754 255 CpG sites in the cord blood of 230 Japanese women and showed no significant differences between women with GDM compared to women without GDM (50). The identification of differentially methylated genes in peripheral and cord blood provides potential for DNA methylation as a biomarker of GDM. Although further validation using pyrosequencing and conducting longitudinal studies in large sample sizes and in different populations are required to investigate their candidacy as biomarkers of GDM.

## Discussion

The identification of dysregulated DNA methylation patterns may aid in elucidating the pathophysiological mechanisms that link maternal diabetes with pregnancy complications and adverse maternal and infant health outcomes. This review aimed to summarize and synthesize studies that have profiled DNA methylation in pregnancies complicated by T1DM, T2DM and GDM. The 32 studies included in this review investigated GDM or IGT and identified a total of 62 genes associated with these disorders. Eight genes including *ADIPOQ, HIF3α, IL-10, LEP, MEG3, PGC-1α, PPARα* and *SNRPN* were differentially methylated in women with GDM or IGT compared to women with normoglycemia in two or more studies. Of these, three genes, *HIF3α*, *PGC1-α* and *PPARα* were similarly differentially methylated in two or more studies. *HIF3α* and *PGC1-α* were hypermethylated, while *PPARα* was hypomethylated in women with GDM compared to pregnant women with normoglyceamia. The consistent methylation profiles of these genes across diverse populations with varying pregnancy durations, and using different diagnostic criteria, methodologies, biological material, support their candidacy as biomarkers of GDM.

Despite our search identifying 32 articles on DNA methylation profiling during maternal diabetes, none of the identified studies profiled DNA methylation in pregnant women with T1DM and T2DM. A study by Alexander et al. profiled DNA methylation in placental tissue of women with GDM (n=14) and pre-existing T2DM (n=3). However, this study correlated DNA methylation with offspring sex and did not compare DNA methylation across diabetes groups, therefore was not included in this review (97).

None of the four studies that measured global DNA methylation reported consistent associations between global DNA methylation and GDM (26, 30, 43, 53). Global DNA methylation profiling provides a robust, relatively easy and cost effective measure of assessing overall genomic DNA methylation (43). However, it may not offer the resolution required to detect CpG specific methylation, which may be more relevant to regulating the expression of genes that orchestrate the development of disease. Thus, studies using methods such as bisulfite pyrosequencing, methylation-specific PCR, methylated DNA immunoprecipitation (MeDIP), direct methylation sequencing and target sequencing combined with base-specific cleavage to assess gene-specific methylation, provided more informative data about the pathogenesis of GDM, and are discussed below.

Of the 62 genes associated with GDM and IGT, eight genes were investigated in two or more studies, of which the increased methylation of *HIF3α* and *PGC1-α* and the decreased methylation of *PPARα* in women with GDM compared to women without GDM were consistent across studies in diverse populations, using different measurement platforms and methodologies, biological material and diagnostic criteria, supporting their involvement in GDM. The results reported in this review showed that CpG islands in the promoter region of *HIF3α* were more methylated in women with GDM compared to women without GDM in European and Chinese populations (23, 46), and that higher methylation was correlated with decreased *HIF3α* gene expression (23). Hypoxia-inducible factors (HIFs) are transcription factors that mediate hypoxia in many tissues (98) and HIF3α has been shown to play a role in glucose metabolism, adipocyte differentiation and inflammation, which are important pathways associated with GDM (68, 99). Previous studies have reported that increased *HIF3α* promoter methylation is associated with obesity and higher plasma glucose levels and waist-hip ratio (71, 100) and adipose tissue dysfunction, T2DM and lower insulin sensitivity (70, 71, 101). Moreover, increased methylation of *HIF3α* in umbilical cord tissue was associated with higher gestational weight gain, infant birth weight and adiposity (102), suggesting that *HIF3α* methylation may be a potential biomarker from metabolic syndrome. In contrast, a recent study reported an association between decreased *HIF3α* methylation and pre-eclampsia (98), a pregnancy complication often leading to placental hypoxia (103). Given the association between GDM, pre-eclampsia and adverse maternal and offspring health outcomes, future studies to determine the functional significance of increased *HIF3α* methylation during GDM, and its relevance to subsequent health outcomes, are warranted.

Three studies reported increased *PGC1-α* methylation in the placentae of women with GDM compared to pregnant women with normoglycaemia in Canadian (39) and Chinese populations (34, 55). These results are aligned with previous findings of increased *PGC1-α* promoter methylation in skeletal muscle biopsies and pancreatic islets of patients with T2DM compared to normoglycemia, which were associated with decreased *PGC1-α* expression and insulin secretion (104, 105). *PGC1-α* is a transcriptional regulator of oxidative metabolism and is critical for fatty acid oxidation and mitochondrial biogenesis (106). Dysregulated mitochondrial function in placenta is thought to play a key role in the pathogenesis of GDM and its complications (107). Thus, increased *PGC1-α* promoter methylation and subsequent decreased *PGC1-α* expression may lead to mitochondrial dysfunction, oxidative stress, insulin resistance and GDM. Interestingly, increased placental *PGC1-α* methylation has been linked to low infant birth weight, supporting the relationship between *PGC1-α* methylation and fetal programming (108).

Two studies reported decreased DNA methylation of *PPARα* in placentae of women with GDM compared to pregnant women with normoglycaemia in German and Chinese populations (30, 54). In contrast to these findings, a recent study reported a positive association between increased *PPARα* methylation and metabolic syndrome, higher triglyceride levels and homeostasis model assessment of insulin resistance (HOMA-IR) (109). Similarly, studies conducted in animal models reported that increased *PPARα* promoter methylation was associated with decreased *PPARα* expression, insulin resistance and hyperlipidemia (110) and obesity (111) in offspring exposed to unhealthy diets during gestation. These findings allude to the important role of *PPARα* methylation in metabolic disease. PPAR*α* is a transcription factor that regulates a variety of processes including fatty acid oxidation, inflammation and hepatic glucose production (86, 112). Thus, decreased placental DNA methylation of *PPARα* may be a compensatory mechanism to increase glucose and lipid metabolism during GDM. Further mechanistic studies are required to elucidate the role of *PPARα* methylation in the pathophysiology of GDM.

Genome-wide methylation was conducted in 12 out of the 32 studies included in this review. The number of differentially methylated CpG sites ranged between 27 and 6892, targeting several genes. The data filtering criteria used for BeadChip array analysis varied significantly across studies. For example, eight of the 12 studies used the more stringent multiple testing correction methods with adjusted *p*-values <0.05 for their analysis (28, 31, 36, 40, 45, 46, 48, 50), whereas four studies used the unadjusted *p-*value of either < 0.01 or < 0.05, since the multiple testing correction methods do not always identify significant differentially methylated loci when sample sizes are small (33, 42, 56, 57). Small sample sizes has been identified as a major limitation of genome-wide studies, which can be improved by using additional datasets and independent DNA methylation quantification methods to verify and validate study findings, although this is time consuming and costly. Indeed, only three of the 12 studies quantifying DNA methylation using BeadChip arrays verified their findings within the same study and confirmed their findings for the relevant genes of interest (36, 40, 57). Other technical differences which may influence the number of differentially methylated CpG sites identified across studies include sample preparation, loading during hybridization and batch effect bias (96, 113). These differences make it difficult to compare DNA methylation profiles across studies and has been identified as a major bottleneck for the identification of robust DNA methylation associations.

The variation in DNA methylation profiles across the studies included in this review, highlight key challenges that must be addressed before DNA methylation profiling can achieve clinical applicability. DNA methylation heterogeneity is attributed to differences in methodology, measurement platforms, normalization strategies, biological source, diagnostic criteria, and timing of methylation analysis (114, 115), thus, emphasizing the need to implement common practices and standardization of experimental approaches to facilitate reproducibility and data harmonization across studies. Studies included in this review utilized various biological material, such as placental tissue, adipose tissue, cord blood and peripheral blood, which consist of a heterogenous mixture of different cell types each possessing a unique DNA methylation signature that may have contributed to a disunited DNA methylation signal observed across studies (114).Of the 32 studies, only three studies (30, 42, 46) adjusted for cell type composition, while no studies reported utilizing the cell-sorting method to measure DNA methylation. Future DNA methylation studies should consider purification of blood cell populations to separate specific cell types, or using bioinformatic methods to adjust for cell type proportions (116). Other factors widely reported to affect DNA methylation heterogeneity include population differences such as age, ethnicity, socioeconomic status, environmental and lifestyle factors such as diet and physical activity, diabetes medication regimes and human immunodeficiency virus (HIV) infections (117–122), that could explain some of the inconsistencies in the findings reported in this review. In addition, pre-analytical factors during sample collection and transport, and analytical factors during DNA isolation and profiling procedures may also contribute the differences in DNA methylation profiling observed in these studies. Furthermore, recent evidence has suggested that DNA methylation may also be influenced by distal genetic sequence variants (123) and in this regard, the use of methylation quantitative trait loci may open exciting opportunities to better understand the complex relationship between genetics, environmental cues, and their impact on the epigenome.

## Conclusion

**Figure 2:**
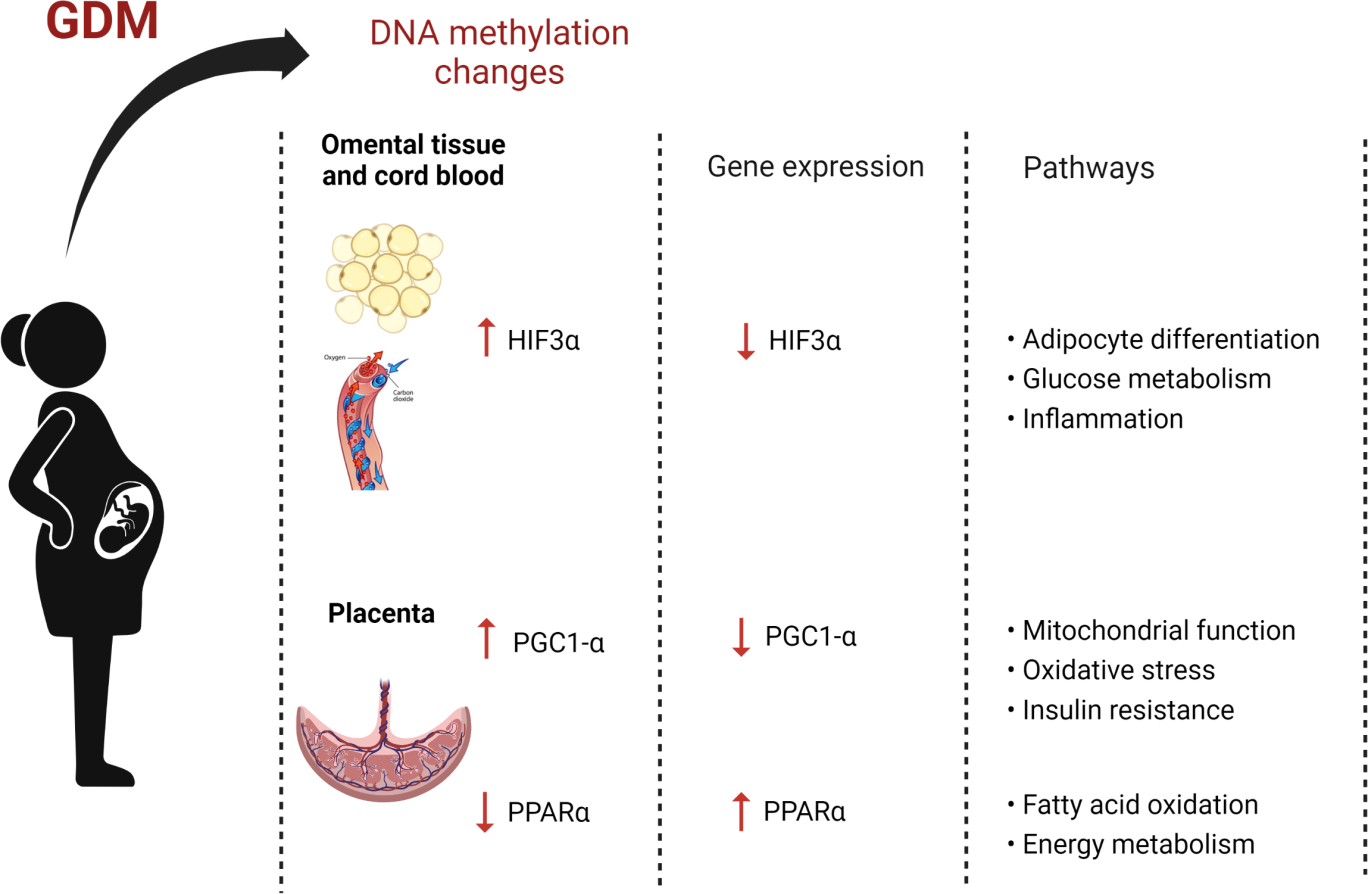
The effects of gestational diabetes on maternal DNA methylation (Image created with Biorender.com).

While a plethora of studies investigated DNA methylation alterations in pregnancies complicated by GDM, our review highlights the lack of studies profiling DNA methylation in pregnancies with pregestational T1DM and T2DM. We propose that future studies should prioritize profiling DNA methylation in pregnancies complicated by the different types of maternal diabetes, to provide insight into their underlying molecular mechanisms, which may be related to pregnancy health outcomes. Furthermore, this review confirms the growing evidence supporting the potential of DNA methylation to serve as biomarkers of GDM. Two genes, *HIF3α* and *PGC1-α*, showing increased methylation and one gene, *PPARα*, showing decreased methylation in women with GDM compared to pregnant women with normoglycemia were consistently methylated across diverse populations with varying pregnancy durations and using different diagnostic criteria, methodologies and biological material. These three differentially methylated genes represent candidate biomarkers for GDM and may influence several GDM-related metabolic processes such as adipocyte differentiation, inflammation, mitochondrial function, oxidative stress, and glucose and energy metabolism (**Figure 2**). Furthermore, these genes may provide insight into the pathways that are epigenetically influenced during diabetes in pregnancy and should be prioritized and replicated in longitudinal studies and in larger populations to ensure their clinical applicability. Profiling DNA methylation may provide an opportunity to facilitate intervention strategies and risk assessment models to identify women at risk of GDM and thus delay or prevent its development and consequent adverse outcomes.

## Author contributions

SD and CP conceptualized the study; SD prepared the original draft; SD, TW, SA, and CP reviewed and edited the manuscript. All authors read and approved the final draft.

## Funding

This work was funded by the National Research Foundation (NRF), Thuthuka Grant No: 129844 to SD and Grant No: 129897 to TW, and the NRF Competitive Programme for Rated Researchers Grant No: 120832 to CP. Baseline funding from the South African Medical Research Council (SAMRC) is also acknowledged.

## Conflict of interest

The authors declare that the research was conducted in the absence of any commercial or financial relationships that could be construed as a potential conflict of interest.

## Publisher’s note

All claims expressed in this article are solely those of the authors and do not necessarily represent those of their affiliated organizations, or those of the publisher, the editors and the reviewers. Any product that may be evaluated in this article, or claim that may be made by its manufacturer, is not guaranteed or endorsed by the publisher.

## Author disclaimer

The content hereof is the sole responsibility of the authors and do not represent the official views of the NRF or SAMRC.
